# Triplexed CEA-NSE-PSA Immunoassay Using Time-Gated Terbium-to-Quantum Dot FRET

**DOI:** 10.3390/molecules25163679

**Published:** 2020-08-12

**Authors:** Shashi Bhuckory, K. David Wegner, Xue Qiu, Yu-Tang Wu, Travis L. Jennings, Anne Incamps, Niko Hildebrandt

**Affiliations:** 1 CEA, CNRS, Institute for Integrative Biology of the Cell (I2BC), Université Paris-Saclay, 91198 Gif-sur-Yvette, France; shashi.bhuckory@protonmail.ch (S.B.); qiuxue@ouc.edu.cn (X.Q.); yu-tang.wu@outlook.com (Y.-T.W.); 2 Federal Institute for Materials Research and Testing (BAM), Division Biophotonics, Richard-Willstaetter-Strasse 11, 12489 Berlin, Germany; karl-david.wegner@bam.de; 3 School of Medicine and Pharmacy, Ocean University of China. 5, Yushan Road, Qingdao 266003, Shandong, China; 4 Thermo Fisher Scientific, 5781 Van Allen Way, Carlsbad, CA 92008, USA; travis.jennings@thermofisher.com; 5 Thermo Fisher Scientific Cezanne SAS, Clinical Diagnostic Division, 30000 Nimes, France; anne.incamps@thermofisher.com; 6 Laboratoire COBRA (Chimie Organique, Bioorganique, Réactivité et Analyse), Université de Rouen Normandie, CNRS, INSA, 76821 Mont-Saint-Aignan, France

**Keywords:** lanthanides, nanoparticles, fluorescence, biosensing, multiplexing, PSA, NSE, CEA

## Abstract

Time-gated Förster resonance energy transfer (TG-FRET) between Tb complexes and luminescent semiconductor quantum dots (QDs) provides highly advantageous photophysical properties for multiplexed biosensing. Multiplexed Tb-to-QD FRET immunoassays possess a large potential for in vitro diagnostics, but their performance is often insufficient for their application under clinical conditions. Here, we developed a homogeneous TG-FRET immunoassay for the quantification of carcinoembryonic antigen (CEA), neuron-specific enolase (NSE), and prostate-specific antigen (PSA) from a single serum sample by multiplexed Tb-to-QD FRET. Tb–IgG antibody donor conjugates were combined with compact QD-F(ab’)_2_ antibody acceptor conjugates with three different QDs emitting at 605, 650, and 705 nm. Upon antibody–antigen–antibody sandwich complex formation, the QD acceptors were sensitized via FRET from Tb, and the FRET ratios of QD and Tb TG luminescence intensities increased specifically with increasing antigen concentrations. Although limits of detection (LoDs: 3.6 ng/mL CEA, 3.5 ng/mL NSE, and 0.3 ng/mL PSA) for the triplexed assay were slightly higher compared to the single-antigen assays, they were still in a clinically relevant concentration range and could be quantified in 50 µL serum samples on a B·R·A·H·M·S KRYPTOR Compact PLUS clinical immunoassay plate reader. The simultaneous quantification of CEA, NSE, and PSA at different concentrations from the same serum sample demonstrated actual multiplexing Tb-to-QD FRET immunoassays and the potential of this technology for translation into clinical diagnostics.

## 1. Introduction

Lanthanide photoluminescence (PL) is implicated in a wide variety of technologies [1], including photovoltaics [2,3], optical imaging [4,5], and biosensing [6,7]. The specific PL features of lanthanide ions are caused by the parity (Laporte) and spin-multiplicity forbidden f–f optical transitions, which give rise to PL spectra with multiple very narrow emission bands in the ultraviolet (UV, for Gd^3+^), the visible (Vis, e.g., for Eu^3+^ and Tb^3+^), and the near-infrared (NIR, e.g., for Nd^3+^ and Yb^3+^) electromagnetic spectrum and extremely long PL lifetimes (up to a few ms) [8,9]. The forbidden transitions also cause extremely low photon absorption cross-sections as expressed by very low molar absorptivities or extinction coefficients (typically below 3 M^−1^ cm^−1^) [10]. Coordination of the luminescent lanthanide ion inside supramolecular chelates or cryptates can enhance the absorption by more than 1000-fold via the antenna effect and efficiently protect the lanthanide ion from the environment to circumvent PL quenching and yield high PL quantum yields [11,12,13,14,15,16,17]. Such bright and stable lanthanide complexes have been used in many different applications concerning highly sensitive, multiplexed, and background-free biological and chemical sensing and imaging [18,19,20,21,22,23]. 

The unique PL properties of lanthanide complexes are ideally suited for their application as donors in Förster resonance energy transfer (FRET) biosensing [24,25,26]. Upon FRET, the long PL lifetime of the lanthanide donor is transferred to the acceptor, and a time-gated PL intensity detection (in a specifically delayed temporal detection window after pulsed excitation) of both donor and acceptor can be used for background-free and ratiometric FRET biosensing [27]. This background-free time-gated detection presents the important advantage over conventional steady-state FRET, which is also ratiometric, but all background from autofluorescence and directly excited acceptor fluorescence interferes with the analyte-specific FRET signals, such that the sensitivity is normally significantly reduced. In particular for Tb, the well-separated emission bands allow for FRET to different acceptors, which enables multiplexed FRET detection of different acceptors with only one type of donor [28,29]. A powerful combination is the utilization of luminescent semiconductor quantum dots (QDs) as FRET acceptors for lanthanide donors [30,31,32]. Despite the strong direct excitation of QDs by any wavelength shorter than their PL bands, the long excited states of lanthanides store the energy until the QDs have decayed back to their ground states and become efficient acceptors for FRET from the lanthanides that remained in their excited states. In principle, the narrow and color-tunable PL emission bands of QDs are well adapted to perform multiplexed lanthanide-to-QD FRET [32,33]. We previously developed five-fold Tb-to-QD FRET multiplexing in a biotin-streptavidin proof-of-concept study [34] and triplexed Tb-to-QD FRET for microRNA quantification [35]. However, the actual application of Tb-to-QD FRET multiplexing in antibody-based immunoassays is significantly more challenging. Although QDs possess superior photophysical properties than fluorescent dyes, the latter are significantly smaller, and the preparation of functional fluorescent dye–antibody conjugates for FRET sandwich immunoassays is simpler [29]. Until recently, we could only realize duplexed Tb-to-QD immunoassays using the epidermal growth factor receptors EGFR and HER2 as model biomarkers [36] and a proof-of-concept study concerning triplexed immunoassays using three QD colors but only a single serum biomarker (prostate-specific antigen, PSA) [37].

Here, we significantly advance multiplexed Tb-to-QD FRET toward real immunoassays by the simultaneous quantification of carcinoembryonic antigen (CEA), neuron-specific enolase (NSE), and PSA from a single serum sample. CEA and NSE are among the tumor markers whose serum levels are monitored in combination with other markers (multiplexing) to obtain a more specific lung cancer diagnosis that can potentially distinguish between small-cell and non-small-cell lung carcinoma [38,39]. PSA was selected as the third tumor marker because of its importance for the characterization and risk assessment of prostate cancer prior to therapy [40,41,42,43] and to demonstrate the possibility of multiplexed quantification of antigens (AGs) with significantly different sizes (approximate values for the carcinoembryonic antigen (CEA): 180 kDa; neuron-specific enolase (NSE): 95 kDa; and prostate-specific antigen (PSA): 32 kDa). A pair of primary IgG antibodies (ABs) against different epitopes of each tumor marker was used for Lumi4-Tb (Tb) donor and QD acceptor conjugation. While the Tb–AB conjugates differed only in the type of IgG ABs (against CEA, NSE, and PSA, respectively), three distinct QD acceptors with respective PL emission maxima at 605 nm, 650 nm, and 705 nm were functionalized with reduced F(ab’)_2_ ABs (from IgGs against different epitopes of CEA, NSE, and PSA) to obtain smaller QD–AB conjugates for improved FRET. To evaluate the pros and cons of multiplexing, we compared (1) single AG detection with a single AB FRET pair, (2) single AG detection with all three AB FRET pairs, and (3) triplexed AG detection with all three AB FRET pairs. The specificity of the monoclonal antibodies circumvented non-specific binding and resulted in negligible biological crosstalk in the multiplexed assay. The efficient spectral separation of the Tb donor and QD acceptors also reduced the optical crosstalk to almost background levels, such that no correction algorithms were required for multiplexed detection. Our results demonstrate that Tb-to-QD FRET can be applied for multiplexed immunoassays for the simpler and quicker quantification of tumor markers at clinically relevant concentrations with reduced sample and reagent volumes.

## 2. Results and Discussion

### 2.1. Tb–AB and QD–AB Conjugates Characterization

#### 2.1.1. Tb–AB Characterization

**Figure 1:**
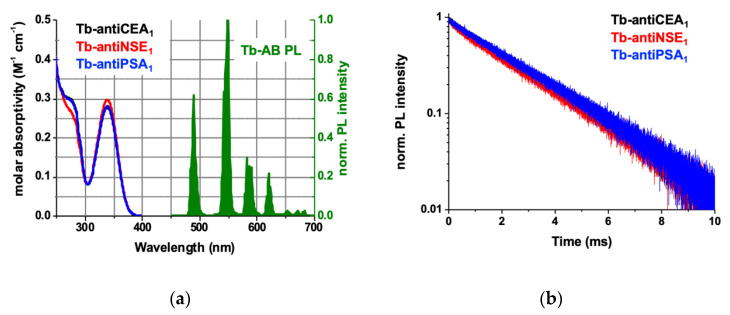
(**a**): Absorption (black, red, and blue lines) and photoluminescence (PL) (green, excitation wavelength: 365 nm) spectra of Lumi4-Tb–antibody (Tb–AB) conjugates. The Tb PL emission lines correspond to the ^5^D_4_ → ^7^F_2-0_ (approximately 650–700 nm), ^5^D_4_ → ^7^F_3_ (approximately 620 nm), ^5^D_4_ → ^7^F_4_ (approximately 590 nm), ^5^D_4_ → ^7^F_5_ (approximately 550 nm), and ^5^D_4_ → ^7^F_6_ (approximately 490 nm) transitions. (**b**): Normalized Tb–AB conjugates PL decay curves upon pulsed excitation at 365 nm with a repetition rate of 100 Hz. Amplitude-averaged decay time: τ(Tb) = 2.6 ± 0.1 ms. Please note that the black curves in (**a**) and (**b**) are almost identical to the blue curves and therefore, they are not very well visible behind the blue curves.

Owing to the small size of Tb compared to a full IgG AB and the sensitivity benefits when many Tb donors surround one QD acceptor [44], Tb was conjugated to full IgG primary antibodies against the different tumor markers. Lumi4–Tb–NHS complexes were directly labeled to available primary amines on the IgG ABs against CEA (antiCEA_1_), NSE (antiNSE_1_), and PSA (antiPSA_1_). Tb-per-IgG conjugation ratios of approximately 10 ± 2 (Tb–antiCEA_1_), 13 ± 3 (Tb–antiNSE_1_), and 10 ± 2 (Tb–antiPSA_1_) were determined by UV-Vis absorption spectroscopy as described in the Methods Section 4.2 and shown in Figure 1a and Table 1. Upon excitation at 365 nm, the PL spectrum of Tb–AB showed the characteristic Tb emission lines around 495 nm, 545 nm, 585 nm, and 625 nm, and between circa 650 nm and 700 nm. The obtained PL decay curves acquired at 490 ± 0.5 nm displayed an almost single exponential and long decay with an average PL lifetime of approximately 2.6 ms (Figure 1b). Notably, the photophysical properties of Tb do not change significantly upon bioconjugation, and the absorption and emission spectra as well as the PL decay of Tb alone (not conjugated to an AB) are almost exactly the same as those shown for the Tb–AB conjugates in Figure 1. The only differences are the AB absorption (below 300 nm) and the PL lifetime, which is 2.7 ms and monoexponential for Tb instead of 2.6 ms and biexponential (very weak fast decay component in addition to the long 2.7 ms lifetime component of Tb) for Tb–AB.

#### 2.1.2. QD–AB Characterization

**Figure 2:**
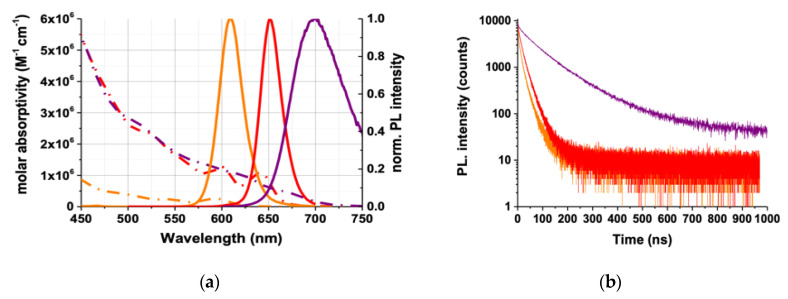

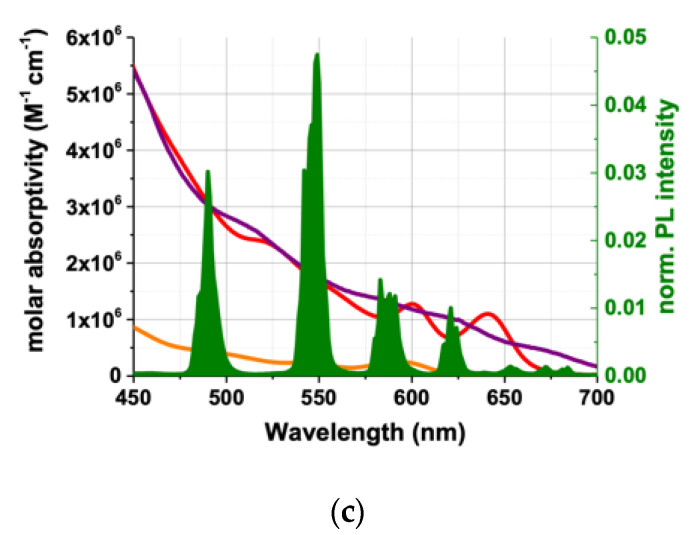
(**a**) Absorption (dash dotted lines) and PL (solid lines) of eQD605 (orange), eQD650 (red), and iQD705 (purple). (**b**) Pulsed (405 nm diode laser at 1 MHz for eQD605/650 and 0.5 MHz for iQD705) excitation was used to measure the PL decay curves at the respective PL peaks of the quantum dots (QDs). The multi-exponential decay curves resulted in amplitude-averaged decay times of τ(eQD605, orange) = 7.4 ± 0.9 ns, τ(eQD650, red) = 17.4 ± 2.5 ns, and τ(iQD705, purple) = 63.5 ± 10.8 ns. (**c**) Overlap between QDs absorption spectra (eQD605: orange; eQD650: red; iQD705: purple) and area-normalized Tb emission spectrum (green). Förster distances: Tb–eQD605 = 7.6 ± 0.4 nm, Tb–eQD650 = 10.8 ± 0.5 nm and Tb–iQD705 = 11.2 ± 0.6 nm.

As a result of the similar sizes of QDs and IgGs, the distance between Tb donor and QD acceptors can become relatively long for FRET (sandwich immunoassay, in which the Tb–AB and the QD–AB bind to different epitopes of the antigen). We reduced primary IgGs to smaller F(ab’)_2_ ABs, which can result in both shorter donor–acceptor distances and increased conjugation ratios of ABs per QD (i.e., increased Tb-donor-per-QD-acceptor ratio) for higher sensitivities [44,45]. All QD–AB conjugations were performed through sulfhydryl chemistry. QD–AB conjugates concentrations were calculated by UV-Vis absorption spectroscopy using the respective molar absorptivities of eQDs (eBioscience, San Diego, CA, USA), iQD705 (Thermo Fisher Scientific, Waltham, MA, USA), and F(ab’)_2_ as described in the Methods Section 4.3 . The calculated concentrations and labeling ratios are summarized in Table 1. Emission spectra and PL decay curves were measured upon excitation at 405 nm using a continuous-wave xenon lamp and a 405 nm diode laser, respectively, and these are represented in Figure 2a,b. The QDs PL spectra show narrow bands with approximately Gaussian shape and a significantly broader full-width-at-half-maximum (FWHM) of iQD705 compared to the eQDs, which is related to their chemical composition (the iQD705 is CdTeSe/ZnS core/shell, whereas the eQDs are CdSe/ZnS core/shell nanoparticles). The three QDs provided sufficiently separated PL bands (which fit in between the Tb PL bands) combined with good spectral overlaps of Tb PL and QD absorption (Figure 2c) for efficient FRET. The PL decays of all QDs were multi-exponential with amplitude-averaged decay times of 7.4 ± 0.9 ns for eQD605, 17.4 ± 2.5 ns for eQD650, and 63.5 ± 10.8 ns for iQD705. The spectral overlap integrals and the Förster distances (*R*_0_) were calculated using the QD molar absorptivity spectra and the area-normalized Tb PL spectrum as described in the Methods Section 4.5. As expected from the spectral overlaps, the Tb–iQD705 FRET pair provided the largest Förster distance (*R*_0_ = 11.2 ± 0.6 nm), followed by the Tb–eQD650 FRET pair (*R*_0_ = 10.8 ± 0.5 nm) and the Tb–eQD605 FRET pair (*R*_0_ = 7.6 ± 0.4 nm).

### 2.2. FRET Immunoassays

The clinical cut-off levels (tumor marker concentrations above which the probability of a cancer is increased) of CEA, NSE, and PSA were found to be 5 ng/mL (approximately 28 pM) [43], 12.5 ng/mL (approximately 132 pM) [46], and 4 ng/mL (approximately 125 pM) [41], respectively. Our FRET pairs (with eQD605 for NSE, eQD650 for CEA, and iQD705 for PSA) were selected based on our previously proof-of-concept results [37]. In a homogeneous FRET sandwich immunoassay, donor and acceptor AB conjugates are present at constant concentrations in solution, and FRET cannot occur because the ABs do not interact. The FRET signal increases with increasing antigen (AG) concentration because more and more “donor–AB–AG–AB–acceptor” sandwich complexes are formed, which brings more and more donors and acceptors in close proximity for FRET. When the AG concentration exceeds the AB concentration, the FRET signal levels off at a constant intensity because more AGs do not result in more sandwich complexes (no more available ABs). If the AG concentration is multiple times higher than the AB concentration, the FRET signal even decreases, because “donor–AB–AG” and acceptor–AB–AG” complexes will be formed instead of FRET sandwich complexes. Due to the shape of the assay calibration curve (increase, constant, decrease), this effect is also called the “hook effect”. AG concentrations can only be reliably quantified with high sensitivity in the increasing part of the calibration curve. Since the steepness and the extent (dynamic range) of this increasing part depend on the AB concentrations, they need to be carefully selected. The limit of detection (LoD) does not only depend on the AB concentration but also on the FRET pair (e.g., FRET efficiency, brightness, signal-to-noise ratio), the AB–AG binding affinity and selectivity, and the surrounding environment, which means that the LoD cannot be predicted. Considering our experience with previous Tb-to-QD immunoassays [37,44,45], a low picomolar LoD (able to detect concentrations below and above the clinical cut-off levels) would require low nanomolar concentrations of donor and acceptor ABs. Therefore, we used a constant concentration of 1.5 nM for all QD-F(ab’)_2_ conjugates and Tb–antiCEA_1_ and Tb–antiNSE_1_. For Tb–antiPSA_1_, we decided to use a constant concentration of 3 nM because the PSA is significantly smaller than the NSE and CEA and because the F(ab’)_2_-per-iQD705 labeling ratio was also significantly higher (cf. Table 1). Within all FRET assays, 50 µL of each donor–AB and acceptor–AB (at the concentrations given above) were mixed with 50 µL of the AG prepared in serum, with a total working volume of 150 µL. All AG concentrations shown in the manuscript are those in the 50 µL serum samples unless it is stated otherwise. All assays were measured on a B·R·A·H·M·S KRYPTOR compact PLUS fluorescence immunoreader (Thermo Fisher Scientific) and used TG-FRET detection, which simultaneously acquired the TG PL intensities of the Tb donor (494 ± 10 nm) and the QD acceptor (607 ± 4 nm for eQD605, 660 ± 7 nm for eQD650, and 707 ± 8 nm for iQD705) in a time-window from 0.1 to 0.9 ms after pulsed excitation with a nitrogen laser (337.1 nm, 20 Hz).

#### 2.2.1. Quantitative Detection of Single Tumor Markers

**Figure 3:**
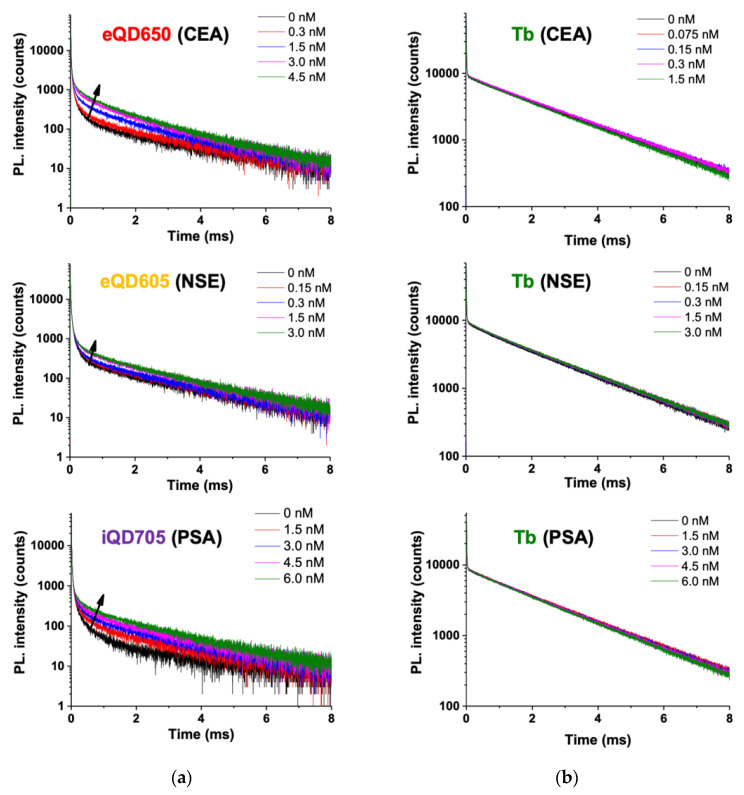
PL decay curves of (**a**) QD acceptors and (**b**) Tb donors with increasing concentration of carcinoembryonic antigen (CEA), neuron-specific enolase (NSE), and prostate-specific antigen (PSA), respectively. Black curves represent the Förster resonance energy transfer (FRET) pairs in the absence of antigens (AG). Very short decay components (in the µs range) arise from the direct excitation of QDs (visible only in (**a**)), and very long decay components (in the ms range) arise from unquenched Tb (visible in both (**a**) and (**b**)). With increasing AG concentrations, a new FRET decay component (in the tens to hundreds of µs range) can be observed with increasing intensity (as indicated by the black arrows in (**a**)).

We first tested the FRET immunoassays for each tumor marker alone to provide a benchmark against which the multiplexed assays can be evaluated. Before the actual assays, we analyzed the PL decays of the QD acceptors and the Tb donor (Figure 3), which clearly showed AG concentration-dependent FRET sensitization of the QDs (Figure 3a) and almost no quenching of the Tb PL (Figure 3b). This stable Tb PL intensity was caused by the relatively large fraction of Tb that did not transfer their energy to QD (Tb–IgGs that are free in solution and IgG-conjugated Tb that are too far away from the QD surface) and is favorable for multiplexed ratiometric detection (FRET ratio: TG PL intensity of QD divided by the TG PL intensity of Tb; Equation (2) in Section 4.6), because the Tb PL intensity will be primarily influenced by the environment (properties of the serum, excitation intensity, etc.) and not by FRET. Thereby, ratiometric TG–FRET leads to higher precision and accuracy (low coefficients of variation) independent of the environmental conditions (both QD and Tb PL are influenced equally, which cancels out in the numerator and denominator). In addition to the long PL decay background of Tb (caused by optical crosstalk of Tb PL in the QD detection channels), the QD PL decays show a new shorter decay component (in the tens to hundreds of µs range) caused by Tb-to-QD FRET upon the formation of Tb–AB–AG–AB–QD sandwich complexes. The intensity of this shorter FRET PL decay component increased with increasing AG concentrations (as indicated by the black arrows in Figure 3a).

**Figure 4:**
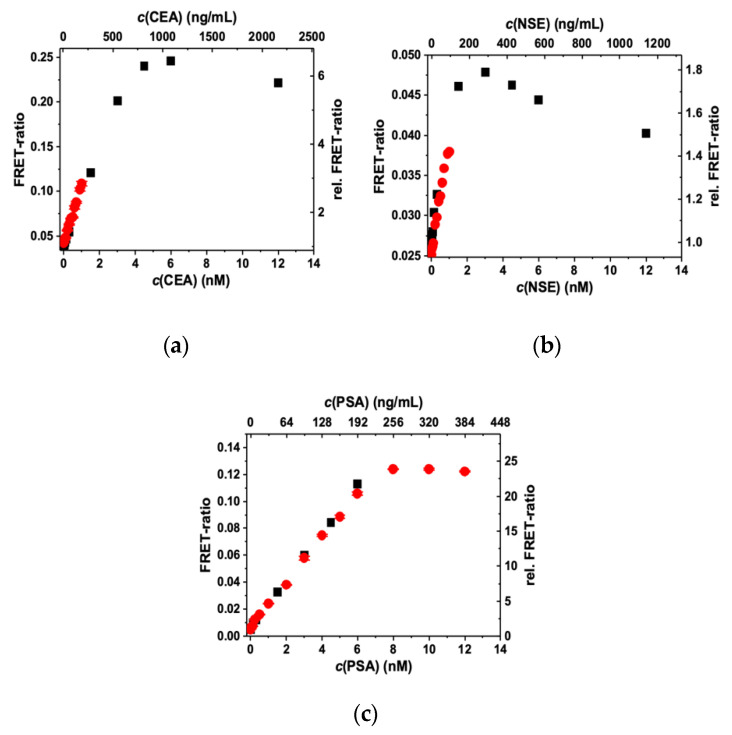
FRET ratios and relative FRET ratios (normalized to unity at 0 nM antigen concentration) of TG QD and Tb PL intensities (0.1 to 0.9 ms after pulsed excitation) as a function of antigen concentration. (**a**) Tb–eQD650 FRET pair for the detection of CEA; (**b**) Tb–eQD605 FRET pair for the detection of NSE; and (**c**) Tb–iQD705 FRET pair for the detection of PSA. The red data points contain more measurements at low concentrations and were used for the determination of limits of detection (LoDs) (cf. Table 2). Only the linear part of the red data points in (**c**) was used for LOD calculation.

The antigen-dependent FRET ratios can be used to record immunoassay calibration curves. The calibration curves shown in Figure 4 correspond to immunoassays with one FRET pair and one AG at increasing concentrations. All three assays exhibit a significant FRET ratio increase with increasing AG concentration due to FRET sensitization of the QDs by Tb donors. Owing to the different FRET pairs and AB concentrations, the dynamic ranges differ for the three assays. For CEA and NSE, the assay curves increase from approximately 10 pM to 4 nM (for CEA) or 2 nM (for NSE). For PSA, the dynamic range spans from approximately 5 pM to 6 nM. These differences were expected due to the higher Tb–IgG concentration and AB-per-QD conjugation ratio for PSA, for which theoretically the curves should start to level off at 1.5 nM for CEA and NSE and at 3 nM for PSA (vide supra). When considering that the differences in FRET pairs, antigen sizes, and conjugation ratios also influence the calibration curves, the expected and experimentally determined concentrations are in very good agreement. For CEA and NSE, one can also see the hook effect (decreasing FRET ratios at very high AG concentrations), whereas PSA only shows the constant FRET ratio region (because of the higher AB concentrations in the PSA assay). The different calibration curves also show that the sensitivity (slope of the linearly increasing parts) is the highest for PSA (relative FRET ratio increase until saturation > 20), followed by CEA (relative FRET ratio increase until saturation approximately 6), and finally NSE (relative FRET ratio increase until saturation approximately 1.7). This difference can be explained by the different FRET pairs because iQD705 (PSA) has the highest signal-to-background ratio (negligible optical crosstalk from Tb PL) and the longest Förster distance, whereas eQD605 (NSE) has a significantly shorter Förster distance and is less bright than eQD650 and iQD705. LoDs for the three assays were determined by repeating the assays with many more different concentrations in the linearly increasing low AG concentration range of the calibration curves (red data points in Figure 4). LoDs were calculated by dividing three standard deviations of 30 measurements of samples without AG (zero concentration) by the slope of the linearly increasing parts of the curves (Equation (3) in Section 4.6). The LoDs were all in the low pM AG concentration range and well below the clinical cut-off values of CEA, NSE, and PSA (Table 2).

#### 2.2.2. Quantitative Detection of Multiple Tumor Markers (Multiplexing)

The detection of multiple biomarkers from a single sample in FRET immunoassays requires careful analysis of biological and optical crosstalk. Such control measurements can evaluate the necessity of applying crosstalk correction for better biosensing results [29,47]. 

#### 2.2.3. Biological Crosstalk

**Figure 5:**
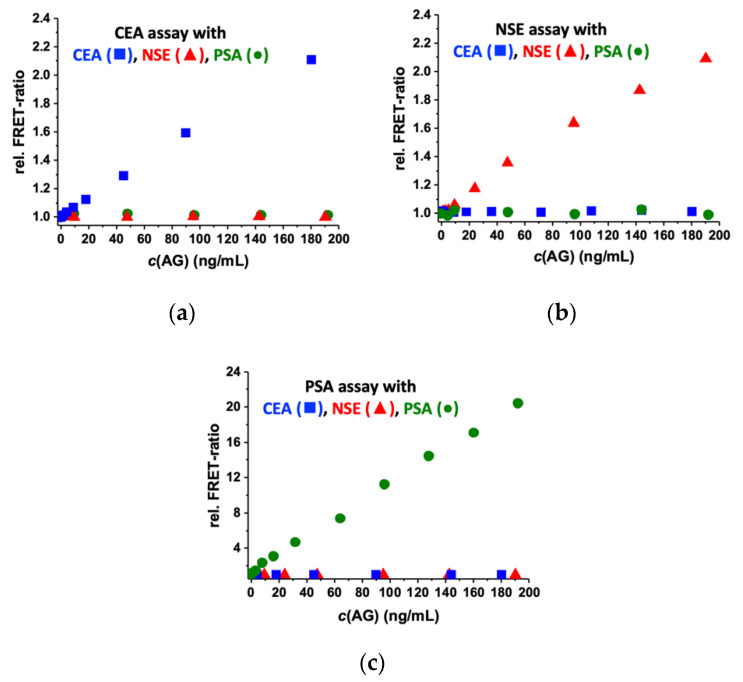
Biological crosstalk evaluation of different AB FRET pairs against different AGs. FRET immunoassay calibration curves for different AG (CEA, NSE, and PSA) concentrations using: (**a**) the antiCEA AB FRET pair measured in the eQD650 detection channel; (**b**) the antiNSE AB FRET pair measured in the eQD605 detection channel; and (**c**) the antiPSA AB FRET pair measured in the iQD705 detection channel.

Biological crosstalk may arise when ABs against a specific AG also bind to other AGs (so-called non-specific binding), leading to a false response of the assay and an over- or underestimation of the respective AG concentrations. To investigate such biological crosstalk, we designed immunoassays to test the specificity of antiCEA AB FRET pairs (in the eQD650 detection channel), antiNSE AB FRET pairs (in the eQD605 detection channel), and antiPSA AB FRET pairs (in the iQD705 detection channel). As shown in Figure 5, only the AB FRET pair-specific AGs resulted in increasing FRET ratios with increasing AG concentrations, whereas the non-specific AGs led to background FRET ratios over the entire concentration range of 0 to 200 ng/mL. These results demonstrate the excellent specificity of the utilized ABs (that are also used in the commercial CEA, NSE, and PSA B·R·A·H·M·S KRYPTOR immunoassay kits of Thermo Fisher Scientific) and that no biological crosstalk exists in the relevant concentration range. Thus, the ABs are ideally suited for multiplexed immunoassays, in which the six different ABs (three AB FRET pairs) are present within the same sample.

#### 2.2.4. Optical Crosstalk

**Figure 6:**
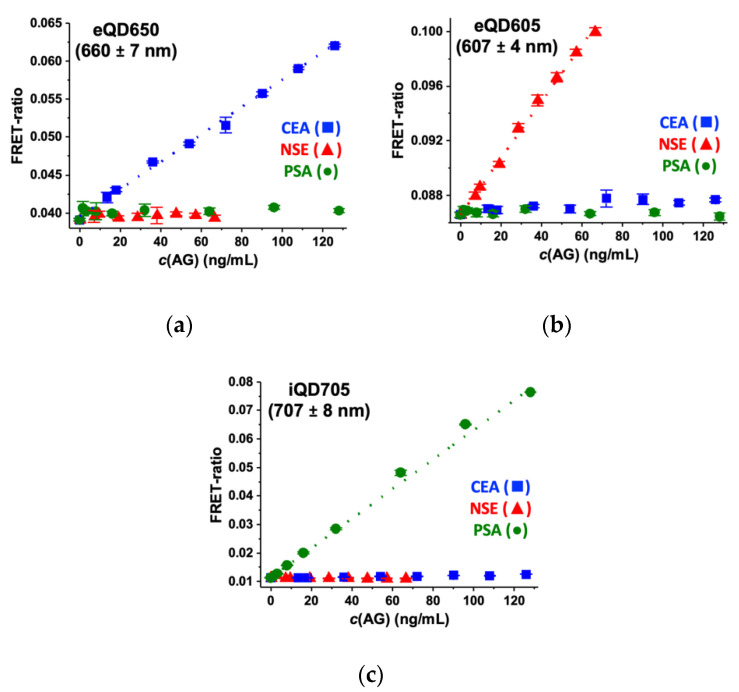
FRET immunoassay calibration curves of CEA, NSE, and PSA (all AB FRET pairs were present in all samples) to determine the optical crosstalk in: (**a**) the eQD650 detection channel for CEA; (**b**) the eQD605 detection channel for NSE; and (**c**) the iQD705 detection channel for PSA. Dotted lines show the linear fits of the AG-specific calibration curves that were used for the determination of LoDs.

Optical crosstalk (or channel bleedthrough) can occur when the PL spectrum of one acceptor is covered by the optical bandpass filter of the detection channel of another acceptor. In those cases, the PL intensity increase of one acceptor would lead to a FRET ratio increase in the detection channels of more than one acceptor, again leading to (usually) positively biased AG concentrations. To evaluate optical crosstalk, we inspected the detection of (1) eQD605 PL in the eQD650 (660 ± 7 nm) and iQD705 (707 ± 8 nm) detection channels; (2) eQD650 PL in the eQD605 (607 ± 4 nm), and iQD705 (707 ± 8 nm) detection channels; and (3) iQD705 PL in the eQD605 (607 ± 4 nm) and eQD650 (660 ± 7 nm) detection channels. Therefore, we devised immunoassays, in which all FRET pairs (antiCEA, antiNSE, and antiPSA ABs) were present, the concentration of one antigen (CEA, NSE, or PSA) was increased, and the FRET ratios were determined from all three QD detection channels (Figure 6). Although all non-specific optical crosstalk signals were extremely low, we observed minor contributions from iQD705 in the eQD650 detection channel (CEA) and from eQD650 in the eQD605 detection channel (NSE). The iQD705 detection channel showed no optical crosstalk at all. The use of spectrally well-separated QD emission bands and narrow optical bandpass filters allowed us to successfully minimize optical crosstalk to almost background levels, which makes the three QD acceptors ideally suited for multiplexed FRET detection. This is remarkable when compared to the optical crosstalk of different dyes acceptors, which require correction for application in multiplexed biosensing [29,47].

The AG-specific calibration curves from Figure 6 could be used to determine the LoDs of the single tumor marker immunoassays in the presence of all AB FRET pairs, which provided a direct evaluation of the influence of crosstalk on assay performance. Compared to the single AB FRET-pair format, the LoDs (Table 3) slightly increased for CEA (1.2 ng/mL to 3.1 ng/mL) and NSE (1.5 ng/mL to 3.1 ng/mL) and remained equal for PSA (0.2 ng/mL). Although the minor optical crosstalk contributions in the eQD650 and eQD605 channels and the lacking crosstalk in the iQD705 detection channel could play a role for the LoDs, the main reason for the LoD increase for CEA and NSE is the 3-fold increased Tb donor concentration (same Tb donor in all AB FRET pairs). Whereas the Tb background PL is extremely low in the iQD705 detection channel, it is significant in the eQD650 and eQD605 detection channels (cf. Figure 3). Thus, the increased Tb background results in slightly more background noise and a less steep slope of the calibration curve. Optimization of the AB concentrations in the multiplexed format may reduce the influence of Tb PL background, but this was out of the scope of our current study. Moreover, the LoDs of CEA and NSE were still below the clinical cut-off levels.

#### 2.2.5. Triplexed CEA/NSE/PSA immunoassay

**Figure 7:**
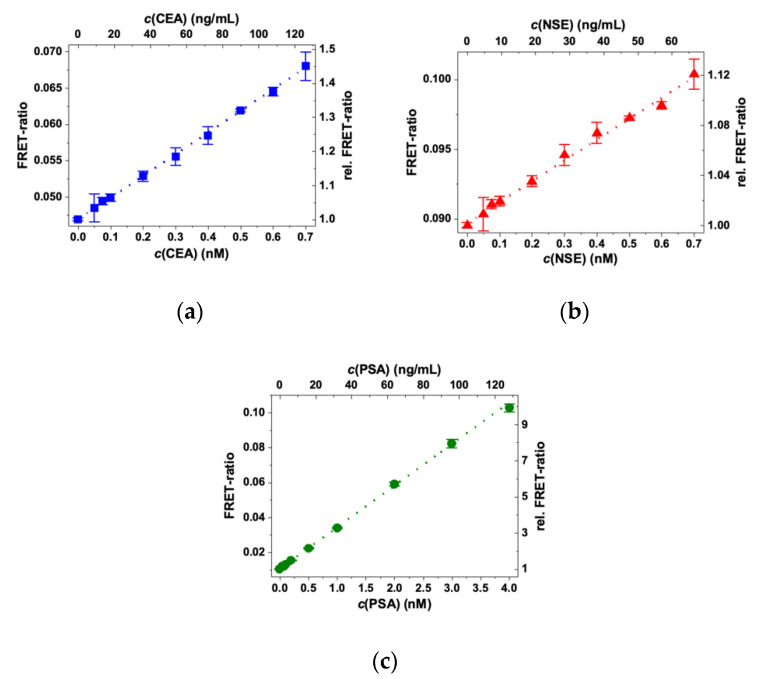
Triplexed FRET immunoassay calibration curves for (**a**) CEA (LoD: 3.6 ng/mL), (**b**) NSE (LoD: 3.5 ng/mL), and (**c**) PSA: LoD: 0.3 ng/mL. Dotted lines show the linear fits of the calibration curves.

Encouraged by the almost negligible optical and biological crosstalks and the low LoDs for the single tumor markers in the multi AB FRET-pair samples, we proceeded to the triplex detection of CEA, NSE, and PSA from a single 50 µL serum sample. In the triplexed immunoassays, the samples with increasing concentrations of CEA, NSE, and PSA were mixed with solutions of 100 µL containing all three FRET AB pairs at constant concentration, similar to those in the single FRET-pair immunoassays. Due to the triplexed format, the overall concentration of ABs was three times higher than in the single tumor marker assays. Despite the presence of six different ABs and three different AGs in the assays, the different FRET ratios specifically increased with increasing concentrations of their respective AGs (Figure 7). The LoDs (Table 3) were only slightly higher than those of the single AG assays with all AB FRET pairs and, with 3.6 ng/mL for CEA, 3.5 ng/mL for NSE, and 0.3 ng/mL for PSA, they were still well below the clinical cut-off levels of the tumor markers.

**Figure 8:**
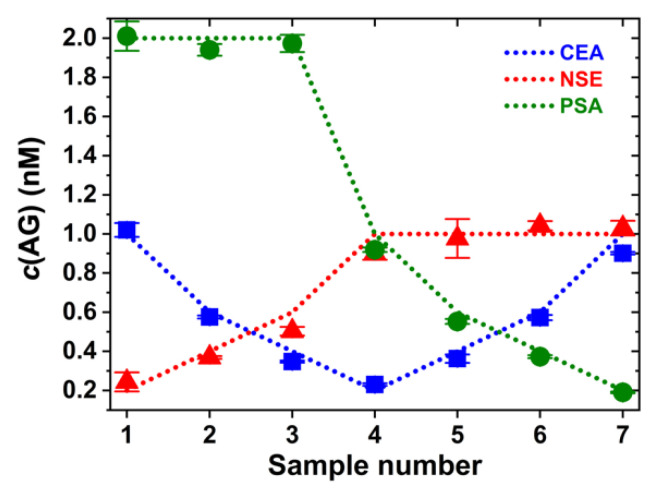
Identification of AG concentrations from samples with different concentrations of CEA, NSE, and PSA by triplexed FRET immunoassay. Dotted lines show the known AG concentrations. AG concentrations of the data points were determined from the triplexed calibration curves in Figure 7. Error bars resulted from triplicate measurements.

Although these triplexed calibration curves already demonstrated the good performance of Tb-to-QD multiplexed immunoassays, the probability of finding different tumor markers at the same concentration within the same sample is quite low. Therefore, we devised a more realistic scenario, in which we challenged our multiplexed FRET immunoassay with a combination of high and low AG concentrations. Seven different samples containing CEA, NSE, and PSA at different and varying concentrations ranging from 0.2 nM to 2 nM were prepared, and the measured FRET ratios were translated into AG concentrations using the calibration curves from Figure 7. The triplexed CEA/NSE/PSA immunoassay (data points in Figure 8) could very well identify the different concentrations (dotted lines in Figure 8 represent the actual concentrations) within the seven different sample compositions with only a few significant deviations and without any biological or optical crosstalk correction.

## 3. Conclusions

Our multiplexed homogeneous FRET immunoassay does not require any washing or purification steps and biological or optical crosstalk correction and can simultaneously detect three significantly differently sized tumor markers from a single serum sample. To experimentally demonstrate the triplexed assay performance, CEA (approximately 180 kDa), NSE (approximately 95 kDa), and PSA (approximately 32 kDa) were precisely quantified by Tb-to-QD FRET probes with one Tb donor (one excitation wavelength for all FRET pairs) and three different QD acceptors (distinguished by different PL emission wavelengths) conjugated to their respective IgG (for Tb) and F(ab’)_2_ (for the QDs) monoclonal ABs. The narrow and Gaussian-shaped emission spectra of the QDs, the well-separated Tb emission bands, and the appropriate optical bandpass filters allowed for negligible optical crosstalk. Thus, no correction was necessary to determine the tumor marker concentrations. All donor and acceptor AB conjugates could specifically bind to their respective AGs, thereby bringing the FRET pairs in close proximity, which resulted in the simultaneous sensitization of all three QD acceptors via FRET from Tb donors that were excited at 337 nm. TG FRET detection (TG window from 0.1 to 0.9 ms after pulsed Tb excitation) was applied to efficiently eliminate the background signals from autofluorescence of the biological samples and direct excitation of the QDs. LoDs in 50 µL serum samples (7 pM or 1.2 ng/mL for CEA, 16 pM or 1.5 ng/mL for NSE, and 6 pM or 0.2 ng/mL for PSA) were below the clinical cut-off levels of all three tumor markers (approximately 5.0 ng/mL CEA, approximately 12.5 ng/mL NSE, and approximately 4.0 ng/mL PSA). The LoDs in the triplexed format (20 pM or 3.6 ng/mL for CEA, 35 pM or 3.5 ng/mL for NSE, and 9 pM or 0.3 ng/mL for PSA) were slightly higher than those in the single AG assays, which means that the benefits of simplicity, speed, and lower reagent and sample consumption come with the cost of slightly reduced assay performance. Nevertheless, the triplexed Tb-to-QD immunoassay could precisely retrieve the AG concentrations from serum samples containing different concentrations of CEA, NSE, and PSA in a 0.2 nM to 2 nM concentration range. Our proof-of-concept study demonstrated the actual multiplexed detection of different clinically relevant tumor markers at clinically relevant concentrations (few ng/mL) and under clinically relevant sample conditions (50 µL serum samples) by Tb-to-QD FRET, which advances this FRET assay technology closer to an actual clinical application. While this step was important for demonstrating the analytical performance of multiplexed TG-FRET with lanthanide donors and QD acceptors, future studies with real clinical samples will be necessary to demonstrate a possible translation to the clinic.

## 4. Materials and Methods

### 4.1. Materials

**Figure 9:**
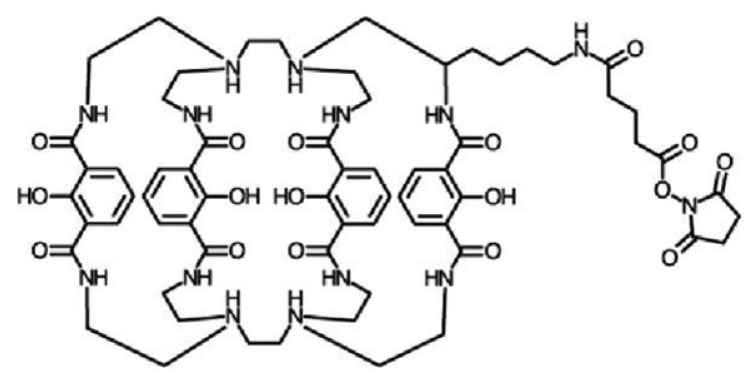
Lumi4 NHS (N-Hydroxysuccinimide) ligand structure (Tb^3+^ ion omitted for clarity).

eFluor quantum dots denoted as eQD605 and eQD650 were provided by eBioscience as part of a sulfhydryl-reactive conjugation kit. Qdot^®^ ITK™ amino PEG 705, referred as iQD705, was purchased from Thermo Fisher Scientific, Waltham, MA, USA. The NHS-activated terbium complex Lumi4-Tb (Figure 9) [16] was provided by Lumiphore in lyophilized form. The tumor markers carcinoembryonic antigen (CEA, approximately 180 kDa), neuron-specific enolase (NSE, approximately 95 kDa), and prostate specific antigen (PSA, approximately 32 kDa), pairs of monoclonal primary antibodies (ABs) against CEA, NSE, and PSA, and bovine calf serum were provided by Thermo Fisher Scientific. IgGs were fragmented to F(ab’)_2_ using a Pierce Mouse IgG F(ab′) F(ab′)_2_ preparation kit and following the instructions provided by the supplier (Thermo Fisher Scientific). Sulfo-EMCS (N-[ε-maleimidocaproyloxy]sulfosuccinimide ester) as well as bovine serum albumin (BSA), tris(hydroxylmethyl)-aminomethane (TRIS/Cl), sodium tetraborate (borate), phosphate buffer saline (PBS), and tris(2-carboxyethyl)phosphine (TCEP) were purchased from Sigma-Aldrich, Saint-Quentin Fallavier, France.

### 4.2. Preparation of Tb–AB Conjugates

Lumi4-Tb–NHS was dissolved to 8 mM in anhydrous DMF and reacted individually in molar excess to available primary amines of the IgG ABs (in PBS) by mixing the solutions in 100 mM carbonate buffer at pH 9. The mixtures were incubated for 2 h at room temperature with rotation at 30 rpm using an ELMI Intelli-Mixer. The IgG–Tb conjugates were purified and washed 4 times with 100 mM TRIS/Cl at pH 7.4 using 50 kDa molecular weight cut-off (MWCO) spin columns from Millipore to remove the unbound Tb complexes. The purified conjugate was stored at 4 °C. Tb concentrations were determined by absorbance measurements at 340 nm using a molar absorptivity of 26,000 M^−1^ cm^−1^ as provided by the manufacturer. ABs were quantified by absorbance measurements at 280 nm using a molar absorptivity of 210,000 M^−1^ cm^−1^ as provided by the manufacturer. The conjugation ratios (Tb per AB) were estimated by linear combination of the respective absorbance values of Tb and ABs within the Tb–AB conjugates.

### 4.3. Preparation of QD–AB Conjugates

IgGs were fragmented to F(ab’)_2_ using a Pierce Mouse IgG F(ab′) F(ab′)_2_ preparation kit following the instructions provided by the supplier. Fragments were verified by SDS-PAGE. eQDs 605 and 650 provided in lyophilized form were reconstituted according to the conjugation kit protocol. The fragmented ABs against CEA and NSE in 1× PBS buffer were mixed directly with eQD650 and eQD605, respectively. The conjugation of F(ab’)_2_ to iQD705 was performed using sulfo-EMCS (a water-soluble heterobifunctional amine-to-sulfhydryl cross-linker that contains NHS-ester on one end and a maleimide reactive group on the other end) where disulfide bonds (S–S) on the F(ab’)_2_ against PSA were reduced to sulfhydryls (S–H) using 5 mM of TCEP (selective reduction of disulfides)in 1 × PBS buffer pH 7.4 with 45 min of incubation with rotation at 30 rpm. Maleimide-activated iQD705 and reduced F(ab’)_2_ were purified twice with 1 × PBS to remove excess sulfo-EMCS and TCEP using 30 kDa MWCO spin columns from Millipore at 1000× *g* and 4000× *g*, respectively. The resulting purified solutions were mixed, and all QD conjugations were incubated for 6 h while rotating at 30 rpm in the dark at room temperature. The QD conjugates were washed 4 times to remove unbound F(ab’)_2_ using a 100 kDa MWCO spin column from Millipore with 100 mM borate buffer pH 8.4 at 1000× *g*, and the purified QD conjugates were further centrifuged at 4000× *g*, from which supernatants were taken and stored at 4 °C. QD–AB conjugates concentrations were calculated by absorbance measurements using molar absorptivities of 2.5 × 10^5^ M^−1^ cm^−1^ (at 594 nm) for eQD605 [48], 1.1 × 10^6^ M^−1^ cm^−1^ (at 641 nm) for eQD650 [48], and 8.3 × 10^6^ M^−1^ cm^−1^ at (405 nm) for iQD705 [49]. F(ab’)_2_ concentrations were determined using a molar absorptivity of 140,000 M^−1^ cm^−1^ at 280 nm. The conjugation ratios (AB per QD) were estimated by linear combination of the respective absorbance values of QDs and ABs within the QD–AB conjugates.

### 4.4. Optical Characterization

Absorption spectra were measured on a Lambda 35 UV/Vis spectrometer from PerkinElmer, Waltham, MA, USA. Photoluminescent (PL) spectra and decay curves were recorded on a FluoTime 300 lifetime fluorescence spectrometer from PicoQuant using as excitation sources a continuous-wave Xe lamp for spectra acquisition, a Xe flash lamp (100 Hz repetition rate) for Tb decay curves, and a 405 nm diode laser (Edinburgh Instruments) for QD decay curves. Tb conjugates and QD conjugates were measured in 100 mM TRIS/Cl buffer pH 7.4 and 100 mM borate buffer pH 8.4, respectively. PL decay curves were acquired directly from the FRET immunoassay samples on a modified B·R·A·H·M·S KRYPTOR compact PLUS plate reader and a prototype Edinburgh Instruments (EI) plate reader using as an excitation source a 20 Hz nitrogen laser operating at 337.1 nm. Spectral separation in the detection channels was performed by optical bandpass filters, where the number after the center wavelength represents the guaranteed minimum bandwidth (GMBW) for Semrock filters (New York, NY, USA) and full-width-at-half-maximum (FWHM) for Delta filters (Hørsholm, Denmark): 494/20 nm (Semrock) for Tb, 607/8 nm (Delta) for eQD605, 660/13 nm (Semrock) for eQD650 and 707/16 nm (Semrock) for iQD705.

### 4.5. FRET Characterization

Förster distances (R_0_, in nm) of the different Tb–QD FRET pairs were calculated using Equation (1) [50,51]:

(1) $$R_{0}=0.021\left(\kappa^{2}\Phi_{Tb}n^{-4}{\left(\left[\int^{}\bar{I_{Tb}}\epsilon_{QD}\lambda^{4}d\lambda \right]\right)}^{1/6}\right)$$

where $\kappa^{2}$ is the dipole-dipole orientation factor of the donor-acceptor FRET-pair, which was approximated as 2/3 for randomly oriented system (dynamic averaging) [50]. $\Phi_{Tb}$ is the Tb-centered PL quantum yield of the Lumi4-Tb donor ($\Phi_{Tb}$ = 0.7) in the absence of the acceptor, and *n* is the refractive index of the medium (*n* = 1.35 for aqueous solutions). The integral function in square bracket was used to calculate the spectral overlap between the Tb emission and QDs absorption, where $\bar{I_{Tb}}$ represents the area normalized (to unity) PL spectrum of Tb in $\lambda^{-1}\;$, $\epsilon_{QD}$ is the molar extinction coefficient spectrum of the QDs in M^−1^ cm^−1^, and λ is the wavelength range of the spectral overlap in nm.

### 4.6. FRET Immunoassays

All FRET assays were measured in black 96-well microtiter plates with an optimal working volume of 150 µL after an incubation time of 180 min at 37 °C. Tb–AB and QD–AB conjugates were each dissolved in 50 µL of 10 mM TRIS/Cl buffer pH 7.4 containing 0.5% of BSA. To this 100 µL solution of Tb–AB/QD–AB, 50 µL of varying concentrations of CEA, NSE, or PSA dissolved in serum were added. All samples were prepared in triplicate except for the antigen-free samples, which were prepared 10 times and measured 3 times. Time-gated (100–900 µs, time window (TW) of 800 µs) and time-resolved PL intensity measurements were acquired on a modified *B·R·A·H·M·S KRYPTOR compact PLUS* plate reader and a prototype EI plate reader, respectively. FRET ratios, proportional to the concentration of the biomarkers, were calculated from the time-gated (TG) PL intensities of Tb and QDs obtained on the KRYPTOR plate reader using Equation (2).

(2) $$FRET-ratio=\frac{I_{TG\left(TW\;800\;µs\right)}\left(QD\right)}{I_{TG\left(TW\;800\;µs\right)}\left(Tb\right)}$$

Limits of detection (LoDs) of the calibration curves were calculated using Equation (3):

(3) $$LoD=\frac{3\times SD}{slope}\;$$

where SD corresponds to the standard deviation of the antigen-free samples (30 measurements), and the slope was determined from the linearly increasing part of the FRET immunoassays calibration curve.

## Figures and Tables

**Table 1:** Concentration of donor and acceptor AB conjugates and their respective conjugation ratios.

Donor-IgG	Tb (µM)	IgG (µM)	Tb/IgG	Acceptor-F(ab’)_2_	QD (µM)	F(ab’)_2_ (µM)	F(ab’)_2_/QD
**Tb-antiCEA_1_**	33.6	3.5	9.7 ± 2.0	**eQD650-antiCEA_2_**	0.48	2.0	4.2 ± 2.0
**Tb-antiNSE_1_**	43.8	3.5	12.5 ± 3.0	**eQD605-antiNSE_2_**	0.20	0.7	3.8 ± 2.0
**Tb-antiPSA_1_**	33.0	2.8	9.9 ± 2.0	**iQD705-antiPSA_2_**	0.19	2.8	14.9 ± 7.0

**Table 2:** Förster distances and LoDs for the single tumor marker (CEA, NSE, and PSA) FRET immunoassays.

Donor–IgG	Acceptor–F(ab’)_2_	Antigen	*R*_0_ (nm)	LoD (nM)	LoD (ng/mL)
**Tb–antiCEA_1_**	**eQD650–antiCEA_2_**	**CEA**	11	0.007	1.2
**Tb–antiNSE_1_**	**eQD605–antiNSE_2_**	**NSE**	8	0.016	1.5
**Tb–antiPSA_1_**	**iQD705–antiPSA_2_**	**PSA**	11	0.006	0.2

**Table 3:** LoDs overview of singleplex, “singleplex”, and triplex format of the FRET immunoassays. All the LoDs are below the clinical cut-off levels: 5.0 ng/mL for CEA, 12.5 ng/mL for NSE, and 4.0 ng/mL for PSA.

Donor–IgG	Acceptor–F(ab’)_2_	Antigen	LoD (nM)	LoD (ng/mL)
**Single-antigen assay with one AB FRET pair**				
Tb–antiCEA_1_	eQD650–antiCEA_2_	CEA	0.007	1.2
Tb–antiNSE_1_	eQD605–antiNSE_2_	NSE	0.016	1.5
Tb–antiPSA_1_	iQD705–antiPSA_2_	PSA	0.006	0.2
**Single-antigen assay with all AB FRET pairs**				
Tb–antiCEA_1_	eQD650–antiCEA_2_	CEA	0.017	3.1
Tb–antiNSE_1_	eQD605–antiNSE_2_	NSE	0.033	3.1
Tb–antiPSA_1_	iQD705–antiPSA_2_	PSA	0.006	0.2
**Triplexed assay (all AGs and all AB FRET pairs)**				
Tb–antiCEA_1_	eQD650–antiCEA_2_	CEA	0.020	3.6
Tb–antiNSE_1_	eQD605–antiNSE_2_	NSE	0.035	3.5
Tb–antiPSA_1_	iQD705–antiPSA_2_	PSA	0.009	0.3
